# Risk assessment of selected bacterial infections acquired in intensive care units in Poland in the years 2019–2023—analysis with the application of the standardized infection ratio

**DOI:** 10.3389/fcimb.2026.1822386

**Published:** 2026-07-30

**Authors:** Monika Filipkowska, Magda Orzechowska, Lukasz Wojcik, Karolina Danilczuk, Mateusz Cybulski

**Affiliations:** 1 Department of Epidemiology, District Sanitary and Epidemiological Station in Bialystok, Bialystok, Poland; 2 Department of Integrated Medical Care, Faculty of Health Sciences, Medical University of Bialystok, Bialystok, Poland; 3 IT Section, Voivodeship Sanitary and Epidemiological Station in Bialystok, Bialystok, Poland; 4 UroClinic-Rogowski, Bialystok, Poland

**Keywords:** antimicrobial resistance, bacterial infections, healthcare-associated infections (HAIs), ICU-acquired bacterial infections, intensive care unit, pathogens, standardized infection ratio (SIR)

## Abstract

**Background:**

Socioeconomic development as well as medical progress has led to a reduction in infectious diseases. The currently observed reappearance of alarming pathogens and the increasing microbial resistance indicate alarming infectious disease resurgence.

**Objective:**

The aim of this study was to evaluate the risk of infections with methicillin-resistant *Staphylococcus aureus* (MRSA), vancomycin-resistant *Enterococcus faecalis* and *Enterococcus faecium* (VRE), *Klebsiella* spp. producing extended-spectrum β-lactamases (ESBL), and *Klebsiella pneumoniae* producing metallo-β-lactamases (MBL) as well as New Delhi metallo-β-lactamase (NDM) in intensive care units (ICUs) in Poland in the years 2020–2023 in comparison to the baseline year of 2019, using the standardized infection ratio (SIR).

**Methods:**

The research material comprised aggregated annual epidemiological data gathered by the Chief Sanitary Inspectorate, which covered all ICUs in Poland in the period from 2019 to 2023, including the number of infections diagnosed after 72 h of hospitalization and the number of ICU person-days. SIR was subjected to an analysis relative to the reference year 2019, and the incidence rate was compared between years and voivodeships with application of the Kruskal–Wallis test and *post hoc* analyses.

**Results:**

In the case of MRSA, elevated SIR values were observed in the Lublin, Subcarpathian, and Silesian Voivodeships. In the Lodz Voivodeship, compared to 2019, a sustained risk reduction was detected. The multiple increase in the risk of *E. faecalis* VRE infections in the Lublin Voivodeship was particularly alarming, with SIR values exceeding the reference level several times between 2020 and 2023. With respect to *E. faecium* VRE, the highest and long-term exceedances were noted in the Holy Cross Voivodeship. The most dynamic growth concerned MBL/NDM-producing *K. pneumoniae* on account of the fact that in the Lower Silesian and Silesian Voivodeships, SIR values reached levels several dozen times higher than those noted in the baseline year. The incidence analysis confirmed material differences between voivodeships, albeit the differences between years were significant primarily in the case of MBL/NDM-producing *K. pneumoniae*.

**Conclusions:**

In the years 2020–2023, significant changes in the risk of infection were observed in ICUs in Poland as well as clear regional variations compared to 2019. The largest and most alarming increases concerned infections caused by MBL/NDM-producing *K. pneumoniae*, indicating the need to strengthen epidemiological surveillance and infection control measures.

## 1 Introduction

Socioeconomic development, medical advances, and improved hygiene and sanitation conditions in the 20th century led to a significant reduction in morbidity and mortality, which had been caused by many infectious diseases, as reflected in the classic theory of epidemiological transition (Omran, 1971). For a considerable period, the prevailing belief was that infectious diseases would cease to pose a significant public health threat in highly developed countries. However, at the turn of the 20th and 21st centuries, infectious diseases regained importance as new pathogens with high epidemic and pandemic potential emerged. This prompted researchers to supplement Omran’s classic concept with a fifth phase of epidemiological transition—the return of infectious diseases (Santosa et al., 2014; Mercer, 2018; McArthur, 2019). From an epidemiological perspective, this allows not only to explain the mechanisms of epidemiological transition but also to monitor the emergence of new alert pathogens further to assess the effectiveness of public health measures (Frérot et al., 2018). This phenomenon is a major challenge for modern healthcare systems (Morens et al., 2004).

Alert pathogens, defined as microorganisms capable of rapid spread, high virulence, and substantial social and economic impact, have become a central public health problem (World Health Organization, 2014). These include both the emerging pathogens such as SARS-CoV-2, influenza viruses with pandemic potential, Ebola virus, and Lassa virus, as well as the re-emergent pathogens, including multidrug-resistant bacteria such as carbapenem-resistant *Enterobacteriaceae* and methicillin-resistant *Staphylococcus aureus* (MRSA) (Fauci et al., 2020). The increasing resistance of microorganisms to available antibiotics, inappropriate use of antimicrobial drugs, globalization, urbanization, and climate change favor both the emergence of new strains and the rapid spread of existing alarming pathogens (Tacconelli et al., 2018; Bedford et al., 2019).

In the context of intensive care units (ICUs), which constitute an environment that is particularly susceptible to nosocomial infections due to patient profile, invasive medical procedures, and overwhelming use of antibiotic therapy, assessing the risk of bacterial infections is key for the safety of healthcare provided there (Choiński et al., 2024).

The most notable antibiotic-resistant pathogens found in the hospital environment, particularly in ICUs, include MRSA, vancomycin-resistant *Enterococcus faecalis* (VRE) and *Enterococcus faecium* (VRE), *Klebsiella* spp. producing extended-spectrum β-lactamases (ESBL), and *Klebsiella pneumoniae* producing metallo-β-lactamases (MBL) and New Delhi metallo-β-lactamase (NDM). These microorganisms are the source of severe infections (sepsis, pneumonia, urinary tract infections, skin and soft tissue abscesses); they are also characterized by high mortality and limited therapeutic possibilities (Tenderenda et al., 2023; Montrucchio et al., 2025).

The abovementioned pathogens have been recognized by the World Health Organization (WHO) and the European Centre for Disease Prevention and Control (ECDC) as pathogens of high (MRSA, *E. faecalis* VRE, *E. faecium* VRE) or critical (ESBL-producing *Klebsiella* spp., MBL/NDM-producing *K. pneumoniae*) importance to public health due to their increasing antibiotic resistance as well as their epidemic potential. Monitoring them in ICUs is of utmost importance when assessing patient safety and the effectiveness of measures implemented in order to prevent healthcare-associated infections (HAIs) (World Health Organization, 2017).

Most epidemiological surveillance studies rely on crude incidence rates, which describe infection frequency but do not compare the observed number of infections with the expected number under a standardized baseline. This limitation becomes particularly important when patient exposure changes over time, such as during the coronavirus disease 2019 (COVID-19) pandemic. Most epidemiological surveillance studies rely on crude incidence rates, which describe infection frequency but do not assess whether the observed number of infections differs from that expected after accounting for patient exposure. Consequently, evidence based on standardized measures of infection risk remains limited, particularly for nationwide ICU surveillance in Poland.

Therefore, this study complements conventional incidence analyses with SIR-based risk assessment. We hypothesized that, after standardization for patient-days, the risk of ICU-acquired infections caused by selected multidrug-resistant pathogens differed between 2020–2023 and the pre-pandemic baseline year (2019), with significant regional variation across Polish voivodeships.

The aim of the study was to evaluate the risk of infections with MRSA, *E. faecalis* VRE, *E. faecium* VRE, ESBL-producing *Klebsiella* spp., and MBL/NDM-producing *K. pneumoniae* in ICUs in Poland between 2020 and 2023 through analyzing epidemiological data by means of the standardized infection ratio (SIR). Moreover, the study objective was to identify temporal trends and regional differences in the incidence of these infections, taking into account the division of the country into voivodeships.

The study addressed the following research questions:

1. Were there any material changes observed in individual voivodeships in terms of the risk of MRSA, *E. faecalis* VRE, *E. faecium* VRE, ESBL-producing *Klebsiella* spp., and MBL/NDM-producing *K. pneumoniae* infections in ICUs in Poland between 2020 and 2023, compared to 2019 as the baseline year?
2. Did the risk of MRSA, *E. faecalis* VRE, *E. faecium* VRE, ESBL-producing *Klebsiella* spp., and MBL/NDM-producing *K. pneumoniae* infections in ICUs in Poland differ significantly between 2019 and 2023 and between individual voivodeships?

## 2 Materials and methods

### 2.1 Study design

An analysis of trends in the risk of infections caused by MRSA, *E. faecalis* VRE, *E. faecium* VRE, ESBL-producing *Klebsiella* spp., and MBL/NDM-producing *K. pneumoniae* was carried out in pediatric and adult ICUs in Poland in the period between 2020 and 2023. The year 2019 was used as the baseline year, as it was the last full year of ICU operation before the organizational and epidemiological changes triggered by the COVID-19 pandemic. It was also the first year with uniformly reported data, aggregated by the Chief Sanitary Inspectorate, covering all analyzed voivodeships and including complete patient hospitalization-day data. Using 2019 as the baseline therefore ensured a stable, pre-pandemic reference point, enabling construction of the SIR indicator and reliable assessment of temporal changes in infection risk.

SIR is defined as the ratio of the number of observed infections to the number of expected infections. It is commonly applied in the epidemiological surveillance of HAIs, including in the National Healthcare Safety Network (NHSN) system of the Centres for Disease Control and Prevention. In this study, the number of expected infections was designated on the basis of the frequency of infections with the discussed pathogens in the reference year 2019 and on the basis of the number of ICU person-days in subsequent years of the analysis, without the application of the multivariate regression models used in the NHSN system (Centers for Disease Control and Prevention, 2026). Unlike the NHSN methodology, the SIR used in this study was based on aggregated surveillance data and did not account for differences in patient or hospital characteristics. Therefore, it reflects overall changes in infection risk over time but does not adjust for potential differences in patient populations between years or regions.

The analysis was performed on the basis of annual data aggregated by the Chief Sanitary Inspectorate, covering all ICUs reported in a given year.

### 2.2 Case definition and outcomes

The analysis included MRSA, *E. faecalis* VRE, *E. faecium* VRE, ESBL-producing *Klebsiella* spp., and MBL/NDM-producing *K. pneumoniae* infections diagnosed after 72 h from admission to ICU, i.e., data relevant for assessing the risk of HAIs.

The definition of HAIs in the ICU and the time criterion for identifying infections acquired during hospitalization were adopted in accordance with the ECDC recommendations contained in the HAI-Net ICU protocol, which is the European standard for epidemiological surveillance of infections in the ICUs (European Centre for Disease Prevention and Control, 2025).

### 2.3 Outcome measures

For the baseline year 2019, the baseline incidence of the studied pathogens acquired 72 h after admission to the ICU was calculated as the ratio of the number of recorded cases during this period to the total number of ICU person-days recorded in 2019. This indicator reflects the incidence of MRSA, *E. faecalis* VRE, *E. faecium* VRE, ESBL-producing *Klebsiella* spp., and MBL/NDM-producing *K. pneumoniae* infections in relation to the duration of patient exposure to the ICU admission. Subsequently, the expected number of infections was calculated for each analyzed year with the assumption that the risk level from 2019 would remain the same. The expected number of infections in a given year was determined by multiplying the baseline incidence of the studied pathogen infections in 2019 by the number of ICU person-days in that year. Such a calculated expected infection rate represents the hypothetical number of infections that would be expected in a given year, assuming an unchanged level of infection risk compared to the baseline year.

SIR was calculated as the ratio of the number of observed infections with MRSA, *E. faecalis* VRE, *E. faecium* VRE, ESBL-producing *Klebsiella* spp., and MBL/NDM-producing *K. pneumoniae* after more than 72 h of ICU hospitalization in a given year to the number of infections expected in that year, determined on the basis of the risk from the baseline year 2019. The resulting SIR value constitutes the measurement unit of the actual number of infections with the expected number after standardization for ICU person-days. SIR values >1 were interpreted as an increase in the risk of infection compared to 2019, SIR values<1 as a decrease in risk, and SIR = 1 as indicating no change in risk relative to the baseline year.

SIR metric as presented in the current manuscript was calculated as a simple ratio of observed and expected cases of infections (further broken down by voivodeship and pathogen):


$$SIR=\frac{o}{e}$$

where:

*o* (observed cases) is the raw number of new infections in a given year;

*e* (expected cases) is the number of expected cases in the year, calculated as baseline frequency *R* times person-days *n*.


e=n·R

Here, the baseline frequency *R* is understood as the number of cases in the reference year (i.e., 2019) divided by person-days in the reference year. Therefore, the discussed metric heavily relies on the number of cases recorded in the reference year.

### 2.4 Statistical analysis

The 95% confidence intervals for SIR were calculated assuming a Poisson distribution for the random variable representing the number of new infections in a given year. Furthermore, 95% confidence intervals were calculated using the exact method employing the relationship between Poisson and chi-square distributions:


$$SIR_{\text{lower}}=\frac{\chi_{\alpha /2,\;\;2o}^{2}}{2e}$$

$$SIR_{\text{upper}}=\frac{\chi_{1-\alpha /2,\;\;2\left(o+1\right)}^{2}}{2e}$$

where *SIR*_lower_ and *SIR*_upper_ are, respectively, the lower and upper confidence limits, *a* is calculated from the assumed confidence level (here *α* = 0.05), and $\chi_{\alpha ,\;\;x}^{2}$ is (100*a)th chi-square centile with *x* degrees of freedom.

The results have been presented as SIR with 95% CIs for each year from 2020 to 2023.

Additionally, in order to answer the second research question concerning differences in incidence between 2019 and 2023 and between voivodeships, a comparative analysis of crude incidence rates was performed. Incidence was defined as the number of infections diagnosed after 72 h from admission to the ICU, divided by the number of ICU person-days and multiplied by 100,000 (incidence per 100,000 ICU person-days). Given that incidence was compared at the level of entire voivodeships across multiple years, rather than within individual ICUs as in device-associated NHSN/ECDC surveillance, incidence was expressed per 100,000 person-days, consistent with the convention used in population- and region-level epidemiological surveillance.

The annual incidence rate for a given voivodeship was the unit of the analysis. Due to non-fulfilment of distribution normality assumptions and the small sample size, the non-parametric Kruskal–Wallis rank-sum test was applied in order to assess the differences between more than two groups. The analysis was conducted in two setups: a comparison of incidence rates between voivodeships throughout the study period and a comparison of incidence rates between 2019 and 2023 on a national scale.

In the intervoivodeship analysis, incidence rates from all analyzed years were compared between individual voivodeships, with the null hypothesis assuming no significant differences between regions and the alternative hypothesis assuming a difference in at least one voivodeship. In the interyear analysis, incidence rates were pooled at the national level and compared between 2019 and 2023, with the null hypothesis assuming no significant differences between years and the alternative hypothesis assuming a difference in at least 1 year.

If either test result indicated statistically significant differences (*p* < 0.05), a *post hoc* analysis was performed using Dunn’s test with Bonferroni’s correction to identify the specific voivodeship pairs (intervoivodeship analysis) or year pairs (interyear analysis) responsible for the significant difference. The level of statistical significance was set at *α* = 0.05. Statistical analyses were conducted in the RStudio environment.

## 3 Results

In the years 2020–2023, a decrease in the SIR value for MRSA was observed from 1.19 (1.12–1.27) in 2020 to 0.92 (0.86–0.99) in 2023 and for ESBL-producing *Klebsiella* spp. from 1.07 (1.03–1.10) in 2020 to 0.83 (0.80–0.86) in 2023. Meanwhile, high SIR values were observed for MBL/NDM-producing *K. pneumoniae* [from 2.21 (2.03−2.40) in 2020 to 4.29 (4.04–4.54) in 2023] and increased rates for VRE, especially *E. faecalis* in 2021, when SIR reached a value of 2.25 (2.01−2.51).

The analysis of SIR for MRSA, *E. faecalis* VRE, *E. faecium* VRE, ESBL-producing *Klebsiella* spp., and MBL/NDM-producing *K. pneumoniae* infections in ICUs in Poland between 2020 and 2023 revealed statistically significant changes in risk compared to the baseline year of 2019 across the voivodeships.

In the case of MRSA, clear regional variation was identified with persistently elevated risk in the Lublin, Subcarpathian, and Silesian Voivodeships throughout the study period (SIR significantly >1 in all analyzed years). The highest value was recorded in the Subcarpathian Voivodeship in 2022 (almost four times above the baseline). By contrast, the Lodz Voivodeship showed a sustained and statistically significant reduction in risk relative to 2019 (**Table 1**).

**Table 1:** SIR for the selected infections in ICUs in Poland in the period from 2020 to 2023, by voivodeship.

Pathogen	Voivodeship		Lower Silesian	Kuyavian-Pomeranian	Lublin	Lubusz	Lodz	Lesser Poland	Mazovian	Opole	Subcarpathian	Podlasie	Pomeranian	Silesian	Holy Cross	Warmian-Mazurian	Greater Poland	West Pomeranian	Poland
MRSA	2020	SIR	0.98	0.82	2.33	1.24	0.67	1.05	1.31	0.72	2.14	0.51	1.45	2.1	0.66	3.13	1.21	0.98	1.19
		95% CI	0.81–1.19	0.48–1.29	1.41–3.65	0.94–1.6	0.55–0.82	0.86–1.26	1.12–1.52	0.44–1.1	1.47–3	0.31–0.8	1.05–1.96	1.71–2.55	0.34–1.15	1.85–4.94	1.02–1.43	0.7–1.33	1.12–1.27
	2021	SIR	1.03	0.62	2.37	1	0.41	1.51	1.33	1.56	1.86	1.07	0.78	1.73	1.21	3.67	1.25	1.57	1.21
		95% CI	0.85–1.23	0.35–1.03	1.5–3.55	0.71–1.38	0.34–0.5	1.3–1.75	1.14–1.54	0.97–2.38	1.34–2.51	0.78–1.43	0.5–1.16	1.42–2.09	0.78–1.8	2.3–5.55	1.06–1.45	1.24–1.97	1.14–1.28
	2022	SIR	1.48	0.66	2.46	0.72	0.54	1.35	0.6	0.72	3.77	1.23	0.53	1.71	0.79	1.44	1.23	1.12	1.10
		95% CI	1.26–1.74	0.37–1.09	1.54–3.72	0.46–1.07	0.43–0.66	1.14–1.59	0.48–0.75	0.36–1.29	2.96–4.74	0.88–1.68	0.3–0.88	1.41–2.06	0.45–1.29	0.81–2.38	1.04–1.44	0.83–1.49	1.03–1.17
	2023	SIR	1.16	0.73	1.82	0.56	0.46	0.72	0.57	1.33	2.82	1.16	1.09	1.71	0.71	1.07	1.06	1.3	0.92
		95% CI	0.95–1.4	0.43–1.17	1.06–2.91	0.34–0.88	0.36–0.59	0.57–0.9	0.45–0.72	0.84–2.02	2.07–3.74	0.83–1.59	0.74–1.54	1.4–2.07	0.38–1.21	0.6–1.76	0.89–1.26	0.98–1.7	0.86–0.99
E. faecalis VRE	2020	SIR	1.58	1.04	11.61	0.4	0.61	0.37	1.11	0	1.23	0	4.97	1.93	0.12	2.43	1.19	1.13	1.14
		95% CI	0.92–2.53	0.03–5.8	4.67–23.92	0.11–1.03	0.35–0.99	0.08–1.09	0.65–1.78	NA	0.53–2.42	NA	2.15–9.8	1.33–2.72	0–0.65	0.29–8.78	0.74–1.82	0.23–3.29	0.96–1.35
	2021	SIR	2.83	7.63	26.42	1.66	1.35	2.74	1.4	5.2	0.32	0.7	1.75	1.26	0.86	3.5	4.07	1.62	2.25
		95% CI	1.92–4.01	3.29–15.03	15.9–41.25	0.88–2.84	1.03–1.73	1.79–4.01	0.88–2.12	1.69–12.13	0.07–0.92	0.14–2.04	0.36–5.1	0.84–1.82	0.37–1.69	0.72–10.23	3.24–5.04	0.53–3.78	2.01–2.51
	2022	SIR	2	11.13	19.61	0.85	1.9	1.23	1.45	3.67	0.25	0	1.28	1.73	0.42	3.36	0.74	0	1.59
		95% CI	1.22–3.09	5.56–19.91	10.44–33.54	0.31–1.84	1.44–2.45	0.59–2.26	0.93–2.16	1.0–9.39	0.03–0.89	NA	0.16–4.63	1.23-2.35	0.11–1.08	1.09–7.85	0.41–1.25	NA	1.38–1.82
	2023	SIR	0.9	10.87	28.9	0.98	2.29	2.01	1.98	1.7	1.28	1.79	1.22	2.1	0.93	1.49	0.82	1.51	1.88
		95% CI	0.39–1.78	5.43–19.45	17.65–44.63	0.39–2.01	1.74–2.95	1.15–3.26	1.35–2.82	0.21–6.14	0.59–2.43	0.66–3.89	0.15–4.42	1.54–2.8	0.4–1.83	0.31–4.37	0.47–1.33	0.41–3.87	1.65–2.12
E. faecium VRE	2020	SIR	0.9	0.64	1.92	3.18	0.9	0.83	1.21	0.12	0.76	0.14	1.52	1.35	6.83	0.84	1.44	4.42	1.08
		95% CI	0.65–1.21	0.5–0.82	1.25–2.81	2.09–4.62	0.71–1.11	0.69–1	1.03–1.41	0.02–0.35	0.5–1.12	0.06–0.28	1.05–2.13	1.12–1.61	4.28–10.34	0.52–1.27	1.17–1.76	3.29–5.81	1.01–1.16
	2021	SIR	1.45	1.13	3.03	3.13	0.89	0.64	1.25	0.87	1.92	0.88	2.63	0.95	9.16	0.98	0.94	3.14	1.23
		95% CI	1.13–1.83	0.94–1.34	2.24–4	1.93–4.78	0.75–1.05	0.53–0.78	1.07–1.44	0.42–1.59	1.56–2.35	0.67–1.14	2.01–3.38	0.78–1.14	6.26–12.93	0.65–1.43	0.74–1.18	2.26–4.25	1.16–1.30
	2022	SIR	1.07	1.17	2.08	2.14	0.78	0.58	0.98	1.61	1.17	1.36	2.66	0.98	5.33	1.58	1.54	1.44	1.16
		95% CI	0.79–1.43	0.97–1.39	1.41–2.95	1.14–3.66	0.62–0.96	0.46–0.72	0.83–1.15	0.99–2.45	0.87–1.54	1.05–1.73	2.01–3.45	0.82–1.18	3.21–8.32	1.24–1.98	1.27–1.86	0.84–2.3	1.09–1.24
	2023	SIR	0.89	0.87	2.63	3.25	0.92	0.77	1.02	0.71	1.06	1.59	2.63	1.67	5.56	0.47	1.32	1.4	1.18
		95% CI	0.62–1.23	0.71–1.06	1.89–3.57	1.99–5.02	0.73–1.14	0.63–0.93	0.86–1.2	0.34–1.3	0.75–1.45	1.26–1.98	2.0–3.4	1.44–1.92	3.3–8.79	0.32–0.67	1.07–1.61	0.8–2.27	1.11–1.25
Klebsiella spp. ESBL	2020	SIR	0.84	0.99	2.97	1.19	1	0.92	0.91	0.42	1.39	0.42	1.77	1.25	1.02	0.96	0.82	1.02	1.07
		95% CI	0.72–0.98	0.85–1.14	2.53–3.46	0.94–1.48	0.91–1.08	0.83–1.02	0.83–1.0	0.34–0.52	1.22–1.57	0.29–0.58	1.54–2.03	1.16–1.35	0.88–1.17	0.78–1.16	0.73–0.92	0.88–1.18	1.03–1.10
	2021	SIR	1.05	1.1	3.74	0.97	0.47	0.93	0.86	0.83	1.14	1.92	1.72	0.96	1.22	1.32	1.32	1.01	1.08
		95% CI	0.91–1.2	0.96–1.25	3.28–4.24	0.72–1.27	0.42–0.51	0.84–1.02	0.78–0.95	0.66–1.02	1.01–1.27	1.66–2.21	1.5–1.97	0.89–1.04	1.08–1.39	1.12–1.56	1.21–1.43	0.88–1.16	1.04–1.11
	2022	SIR	1.04	1.37	2.87	0.8	0.47	0.82	0.55	0.31	1.28	1.27	1.53	0.8	0.85	0.63	0.91	0.78	0.88
		95% CI	0.9–1.2	1.21–1.54	2.45–3.33	0.56–1.1	0.42–0.52	0.74–0.92	0.49–0.62	0.22–0.43	1.14–1.43	1.03–1.55	1.31–1.77	0.74–0.87	0.73–0.98	0.52–0.75	0.82–1.01	0.65–0.91	0.85–0.91
	2023	SIR	1.01	0.84	3.32	0.56	0.5	0.79	0.65	0.53	1.38	1.17	1.7	0.77	0.9	0.32	0.75	0.81	0.83
		95% CI	0.86–1.18	0.72–0.98	2.89–3.81	0.37–0.82	0.45–0.57	0.7–0.88	0.58–0.73	0.41–0.68	1.23–1.56	0.95–1.44	1.47–1.94	0.71–0.84	0.77–1.05	0.26–0.4	0.67–0.84	0.68–0.95	0.80–0.86
K. pneumoniae MBL/NDM	2020	SIR	11.69	0.56	6.95	10.82	NA	5.42	1.92	0.72	NA	0.41	NA	31.69	5.59	0.56	2.26	1.58	2.21
		95% CI	7.83–16.78	0.23–1.16	5.38–8.82	7.92–14.43	NA	3.75–7.57	1.63–2.24	0.15–2.09	NA	0.29–0.56	NA	23.28–42.14	3.58–8.32	0.37–0.83	0.27–8.18	0.63–3.25	2.03–2.40
	2021	SIR	66.39	2.2	9.21	19.65	NA	13.68	4.28	2.6	NA	1.52	NA	25.29	4.08	1.81	4.9	14	4.89
		95% CI	56.73–77.22	1.48–3.14	7.54–11.14	15.2–25.0	NA	11.14–16.62	3.85–4.75	0.84–6.06	NA	1.31–1.77	NA	18.71–33.43	2.45–6.37	1.45–2.24	1.59–11.43	10.95–17.63	4.63–5.15
	2022	SIR	50.65	2.57	8.96	0	NA	9.31	2.6	2.75	NA	1	NA	67.51	3.15	0.36	35	6.15	3.70
		95% CI	41.89–60.71	1.77–3.61	7.25–10.95	NA	NA	7.08–12.0	2.28–2.96	1.01–5.99	NA	0.81–1.24	NA	56.64–79.86	1.76–5.2	0.24–0.51	24.09–49.15	4.09–8.89	3.48–3.94
	2023	SIR	66.08	3.95	11.38	1.63	NA	10.97	2.99	1.27	NA	1.01	NA	49.94	4.17	0.2	46.24	11.57	4.29
		95% CI	55.4–78.21	2.95–5.18	9.48–13.55	0.53–3.79	NA	8.52–13.91	2.63–3.38	0.26–3.72	NA	0.82–1.24	NA	40.4–61.05	2.47–6.59	0.12–0.3	33.73–61.87	8.61–15.21	4.04–4.54

CI, confidence interval; NA, not available; SIR, standardized infection ratio.

With respect to *E. faecalis* VRE infections, the Lublin Voivodeship stood out with a persistently and substantially elevated risk throughout 2020–2023, reaching an almost 30-fold increase compared to baseline in 2023 (95% CI excluding 1 in all years). In the remaining voivodeships, risk fluctuated between periods of significant increase and decrease relative to 2019 (**Table 1**).

For *E. faecium* VRE, the most pronounced and sustained increases were observed in the Holy Cross, Lubusz, and Lublin Voivodeships, with the Holy Cross Voivodeship showing the highest values throughout the entire period (up to a ninefold increase in 2021). The Pomeranian and West Pomeranian Voivodeships also showed elevated, though more variable, risk. In other regions, increases were more transient, typically emerging from 2021 to 2022 onward (**Table 1**). In the years 2020–2023, compared to the reference year 2019, a clear variation in SIR values for ESBL-producing *Klebsiella* spp. infections in ICUs was observed between the voivodeships. The Lublin Voivodeship again showed the highest and most consistent risk elevation across all years (up to nearly fourfold above baseline), followed by the Pomeranian and Subcarpathian Voivodeships with persistent, more moderate exceedances. The Podlasie, Greater Poland, and Warmian-Masurian Voivodeships showed transient increases confined mainly to 2021, while the Lodz and Mazovian Voivodeships showed a clear and lasting decline relative to baseline (**Table 1**).

Analysis of SIR for infections caused by MBL/NDM-producing *K. pneumoniae* showed that in some regions, extremely high exceedances of expected values were observed. The Lower Silesian and Silesian Voivodeships showed extreme, persistent risk elevation, with SIR values reaching several dozen-fold above baseline (up to 66-fold in the Lower Silesian Voivodeship in 2021 and 2023). Substantial and sustained increases were also observed in the Lublin, Lesser Poland, Mazovian, Holy Cross, and Kuyavian-Pomeranian Voivodeships, while the Warmian-Masurian and Podlasie Voivodeships showed more moderate or stabilizing patterns after an initial increase (**Table 1**).

SIR values discussed for the selected infections in ICUs in Poland in 2020–2023, broken down by voivodeship, are presented in **Table 1**.

**Figure 1:**
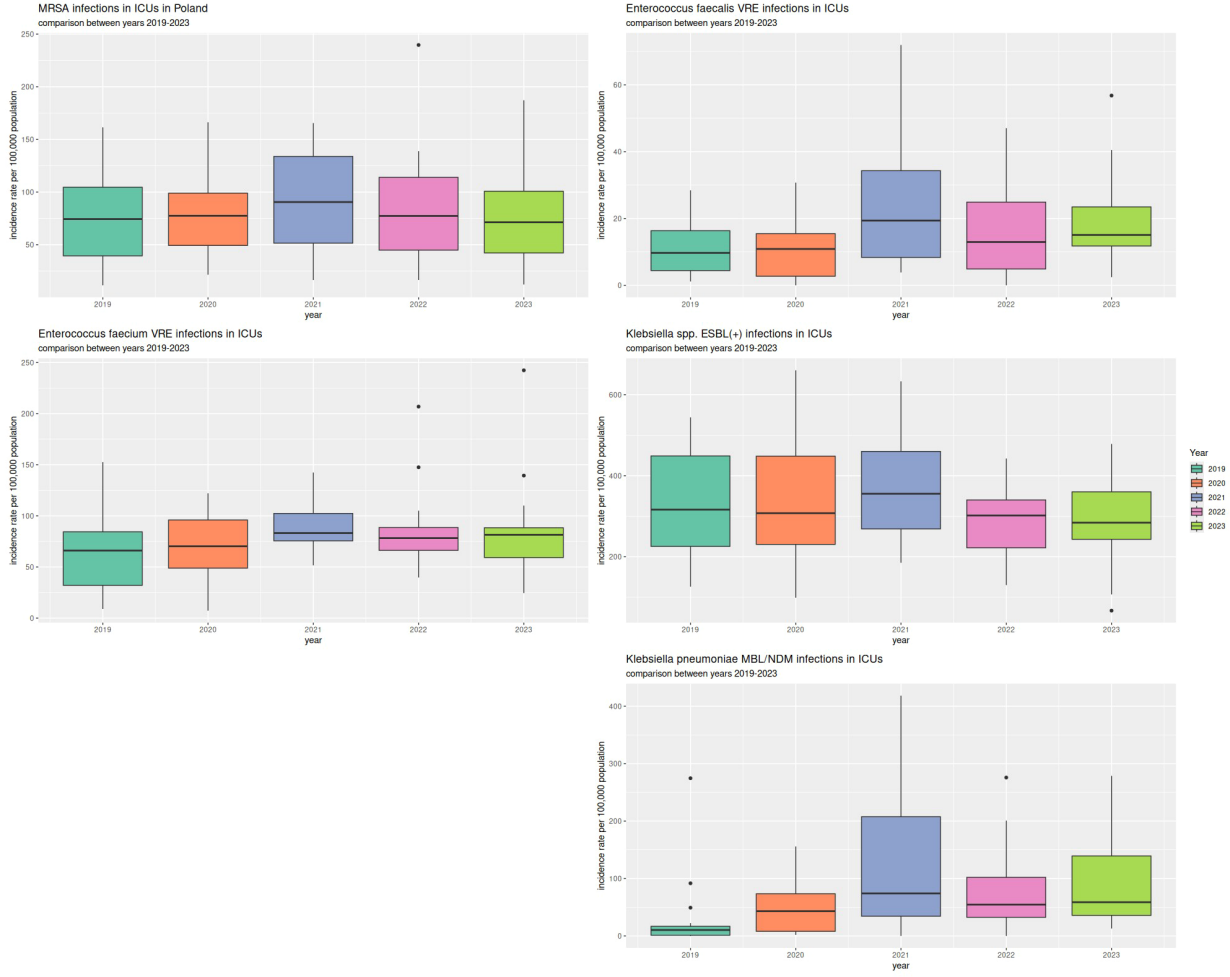
Differences in the incidence of the analyzed pathogens in ICUs in Poland in the years 2019−2023.

**Figure 2:**
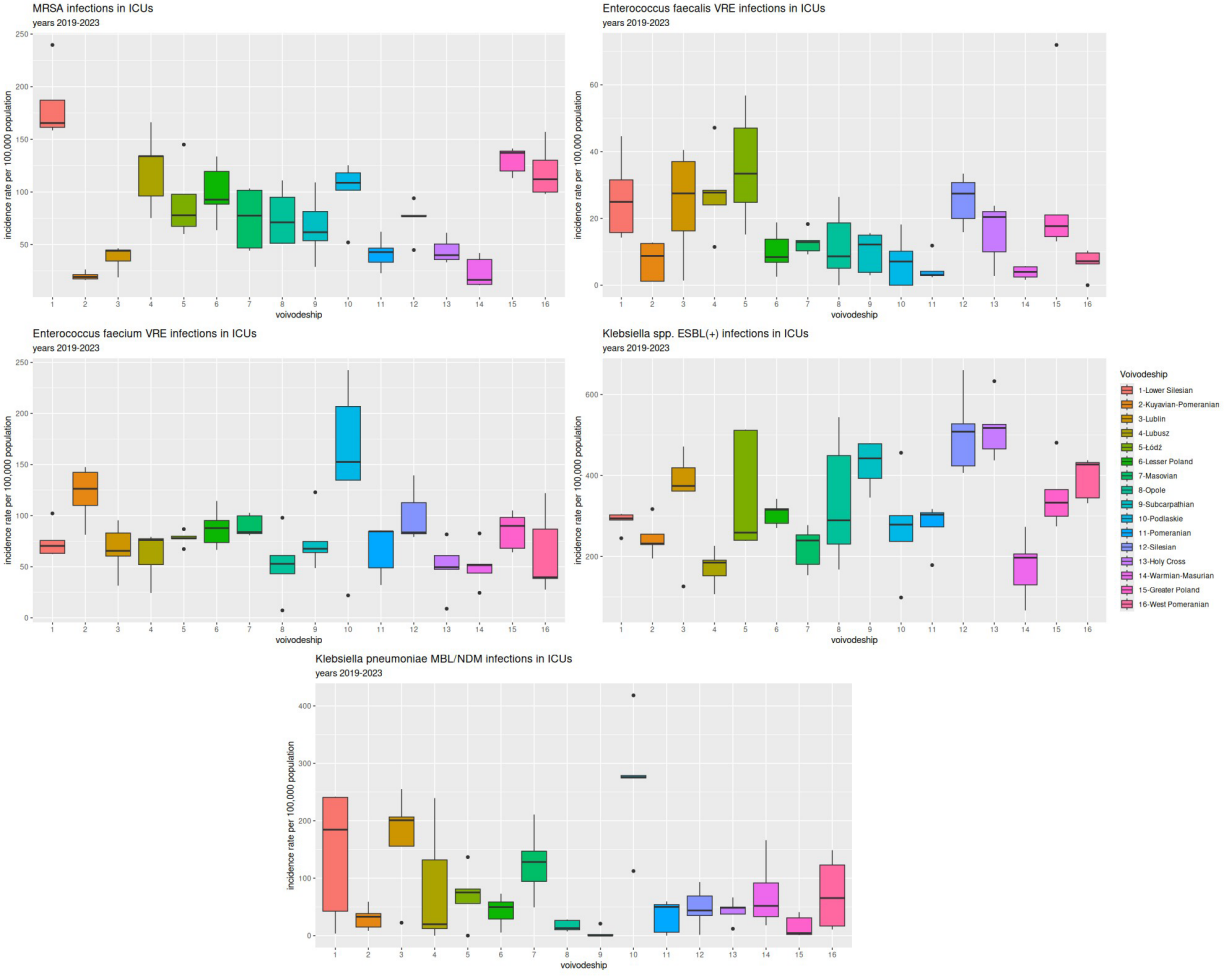
Differences in the incidence of the analyzed pathogens in ICUs in Poland in the years 2019−2023, broken down by voivodeship.

Regional differences in incidence were statistically significant for all five analyzed pathogens (Kruskal–Wallis, *p* < 0.05). At the national level, however, a significant change between 2019 and 2023 was observed only for MBL/NDM-producing *K. pneumoniae* (*p* < 0.05). The remaining pathogens (MRSA, *E. faecalis* VRE, *E. faecium* VRE, ESBL-producing *Klebsiella* spp.) showed no significant nationwide temporal trend (*p* > 0.05). The Lublin, Lodz, Warmian-Masurian, Silesian, Holy Cross, Podlasie, Opole, Subcarpathian, and Greater Poland Voivodeships accounted for most of the significant pairwise differences identified in *post hoc* testing. The differences described in the incidence of MRSA, *E. faecalis* VRE, *E. faecium* VRE, ESBL-producing *Klebsiella* spp., and MBL/NDM-producing *K. pneumoniae* by time and region are graphically presented in **Figures 1** and **2**.

## 4 Discussion

ICUs remain a high-risk area for HAIs, requiring intensive epidemiological surveillance and implementation of advanced preventive measures, indicating the unique importance of enhanced infection control and monitoring of microbial resistance in these hospital units (Filipkowska et al., 2025). It should be emphasized that the present study did not directly measure pandemic-related variables such as antibiotic consumption rates, ICU staffing levels, or specific organizational changes; therefore, the associations discussed below between the COVID-19 pandemic and the observed regional variation in infection risk should be regarded as plausible hypotheses derived from the existing literature rather than as conclusions established by the present data.

The results of the current study clearly show that ICUs constitute an environment that remains at an increased risk of the spread of alarming pathogens, and the years 2020–2023 were characterized by significant dynamic epidemiological changes compared to the baseline year of 2019. Using the SIR index facilitated standardization by the number of hospitalized patient days and the assessment of relative risk in the context of changing patient numbers and the burden on the healthcare system during the COVID-19 pandemic (Weiner-Lastinger et al., 2022). The analyzed period includes years directly related to the SARS-CoV-2 coronavirus pandemic, which significantly changed the functioning of ICUs. Organizational transformations, increased workload on medical staff, staff turnover, changes in patient structure (increased percentage of mechanically ventilated and long-term hospitalized patients), and the widespread use of empirical antibiotic therapy may have promoted the transmission of multidrug-resistant microorganisms. Lee et al. (2023) demonstrated that during the period of greatest strain on the healthcare system, epidemiological surveillance and preventive measures, such as hand hygiene audits and monitoring of contact isolation compliance, were weakened, which correlated with an increase in selected HAI rates, particularly in ICUs. The authors emphasize that the reorganization of ICU work during the crisis involved the creation of *ad hoc* teams to care for COVID-19 patients, often activating staff delegated from other departments and working in new organizational configurations, which could lead to disruptions in the continuity of infection control procedures. As a result, this promoted the transmission of multidrug-resistant microorganisms, especially in the ICU environment, where a high percentage of patients undergoing invasive procedures predominates (Lee et al., 2023).

A systematic review conducted by Abubakar et al. (2023), covering 37 studies from various regions of the world, demonstrated a diverse pattern of changes in overall HAI rates during the pandemic. In some centers, an increase was observed, while in others, no significant differences were found compared to the pre-pandemic period. At the same time, many analyses revealed an increase in the frequency of infections associated with medical devices and an increase in the isolation of Gram-negative bacteria with an MDR phenotype. In particular, a greater proportion of carbapenem-resistant strains and an expansion of the spectrum of β-lactamases, including ESBLs and carbapenemases, were reported (Abubakar et al., 2023).

Regarding Gram-negative bacteria, Al Golli et al. (2025) also described a significant increase in the percentage of MDR strains between 2020 and 2023 compared to the pre-pandemic period. In the analyzed hospital populations, an increased frequency of isolation of *Klebsiella* spp., *Acinetobacter baumannii*, and *Pseudomonas aeruginosa* with a carbapenem-resistant phenotype was observed. The authors indicate that the widespread use of empirical broad-spectrum antibiotic therapy in patients with COVID-19, often without confirmed bacterial co-infection, could have generated significant selective pressure, favoring the elimination of sensitive strains and the expansion of microorganisms with carbapenem resistance mechanisms (Golli et al., 2025). Similar conclusions were presented by Peconi et al. (2025) in a 2025 systematic review of studies analyzing the epidemiological situation during and after the pandemic. The authors noted that most of the included analyses revealed an increase in the frequency of HAIs and an increased share of multidrug-resistant Gram-negative pathogens, especially strains producing ESBLs and carbapenemases. This phenomenon was observed both in ICUs and other hospital wards, suggesting a lasting impact of the pandemic on the structure of microbial resistance (Peconi et al., 2025). In turn, Sakagianni et al. (2025), in a report published in 2025 on the impact of SARS-CoV-2 infections on the epidemiology of resistance in Gram-negative bacteria, emphasized a marked increase in resistance mechanisms, including an increased incidence of carbapenemase production, such as NDM. The authors indicated that the pandemic promoted the spread of strains possessing mobile genetic elements encoding carbapenem-hydrolysing enzymes, which reflects the observed global trends of increased resistance among Gram-negative bacilli (Sakagianni et al., 2025).

The most pronounced and alarming increase was observed in MBL/NDM-producing *K. pneumoniae* infections. Infections caused by carbapenemase-producing *K. pneumoniae* strains are associated with significantly higher mortality, prolonged hospitalization, and increased treatment costs, particularly in the ICU patient population (Martin and Bachman, 2018; Yao et al., 2024). Numerous studies have demonstrated that strains producing NDM and other carbapenemases have a high epidemic potential and the ability to spread, both within wards and between healthcare facilities, which favors the development of nosocomial infection outbreaks. Carbapenem-resistant *Enterobacterales*, including *K. pneumoniae*, are therefore a global epidemiological threat, and their frequency and spread show increasing trends in the period monitored in the study (Caliskan-Aydogan and Alocilja, 2023; Lin et al., 2023; Ma et al., 2025). Reports from the COVID-19 pandemic also indicate an increase in the detection of carbapenemase-producing Gram-negative bacilli in ICUs, an observation that other authors have attributed to an overloaded healthcare system, widespread use of empirical antibiotic therapy, and deficiencies in hospital infection control procedures (Ayoub Moubareck and Hammoudi Halat, 2022); however, these specific mechanisms were not assessed in the present study and remain hypothetical explanations for the regional patterns observed here. Descriptions of NDM-producing *K. pneumoniae* outbreaks in ICUs during subsequent waves of COVID-19 indicate that the pandemic may have been a significant factor contributing to changes in the transmission dynamics of these strains (Amarsy et al., 2021). Healthcare system overload, reorganization of hospital wards, increased antibiotic use, and limited ability to maintain standard infection control procedures may have created conditions conducive to the spread of carbapenemase-producing *Enterobacterales*. In the context of these reports, the multiple exceedances of SIR reference values in 2021 and 2023 observed in this study should be interpreted as a potential indicator of increased regional transmission, requiring the implementation of surveillance based on genetic typing of strains and the strengthening of preventive measures and control of hospital infections.

Some of the observed patterns can be explained by factors extending beyond the COVID-19 pandemic itself. The marked, regionally concentrated rise in MBL/NDM-producing *K. pneumoniae* risk, especially in the Lower Silesian and Silesian Voivodeships, aligns with the well-documented endemic spread of NDM-producing *K. pneumoniae* lineages in Poland, driven primarily by clonal transmission and patient transfers between facilities rather than by pandemic-specific circumstances alone (Biedrzycka et al., 2021). This suggests that the sharp regional increases we observed may reflect the ongoing spread of pre-existing endemic strains in reference hospitals with high patient turnover, further intensified by the additional burden the pandemic placed on infection control systems, including staff shortages, high workloads, ward reorganization, and disruptions to routine hygiene and isolation procedures.

The national decline in MRSA risk, by contrast, may be attributable to the intensified infection prevention measures introduced during the pandemic, particularly enhanced hand hygiene and personal protective equipment use, which have been linked to reduced MRSA acquisition in several healthcare systems worldwide (Shih-Hao et al., 2021; Wee et al., 2021). Since MRSA spreads mainly through direct and indirect contact, it may have responded more strongly to these universal precautions than carbapenemase-producing Gram-negative bacteria, whose persistence depends more heavily on environmental reservoirs and antibiotic selection pressure. This difference may help explain the opposite trends observed for these two pathogen groups in our study.

The regional differences found across all analyzed pathogens may also stem from previously documented variation in regional antibiotic consumption in Poland, which has been linked to demographic, economic, and healthcare organizational factors (Olczak-Pienkowska and Hryniewicz, 2021). Together with the number of high-risk patients requiring invasive procedures and frequent interhospital transfers in certain reference centers, these regional disparities in antibiotic use and patient case-mix likely contribute to the persistent voivodeship-level differences in infection risk, independent of the pandemic period.

### 4.1 Study limitations

When interpreting the obtained results, the following limitations of this study should be taken into account:

1. Inability to control individual patient factors, such as age, comorbidities, duration of mechanical ventilation during hospitalization, use of invasive medical devices (catheterization), and prior antibiotic therapy.
2. Possible differences in the completeness of pathogen reporting to the Chief Sanitary Inspectorate among individual voivodeships.
3. Lack of information regarding the number of ICUs and hospital reference levels, which could not be analyzed in this study.
4. The SIR used in this study was based on aggregated surveillance data and was not adjusted using multivariable regression models, as implemented in the NHSN methodology. Consequently, differences in patient case-mix, severity of illness, hospital characteristics, or other potential confounding factors could not be taken into account.
5. Data on pandemic-related organizational factors potentially influencing regional variation (including antibiotic consumption, ICU staffing changes, and healthcare system overload) were not collected in this study. Consequently, the COVID-19-related explanations proposed in the Discussion are hypotheses consistent with the literature rather than conclusions directly supported by the present data.

## 5 Conclusions

1. Significant changes in the risk of infections with the analyzed pathogens were observed in ICUs in Poland in the period between 2020 and 2023 compared to 2019.
2. The analyses confirmed the existence of clear and persistent regional variations in the risk of infections. Further studies directly measuring pandemic-related organizational and antibiotic use factors are needed to confirm whether they explain these regional differences.
3. The largest and persistent increases in infection risk (SIR > 1 compared to 2019) were observed for MRSA (including in the Lublin, Subcarpathian, and Silesian Voivodeships), *E. faecalis* VRE (particularly in the Lublin Voivodeship), and MBL/NDM-producing *K. pneumoniae* (especially in the Lower Silesian and Silesian Voivodeships).
4. Statistically significant differences were detected in the incidence analysis between individual voivodeships, although no significant differences were found nationally between 2019 and 2023 for most of the analyzed pathogens. MBL/NDM-producing *K. pneumoniae* was an exception with incidence rates significantly higher after 2019 (particularly in 2021 and 2023).
5. The pronounced regional concentration of MBL/NDM-producing *K. pneumoniae* risk likely reflects the spread of pre-existing endemic clones in reference hospitals with high patient turnover, worsened by pandemic-related strain on infection control, whereas the national decline in MRSA risk may reflect the effectiveness of contact-precaution measures intensified during the pandemic.

These findings support stronger antibiotic stewardship and enhanced surveillance in regions with major reference hospitals.

## Data Availability

The raw data supporting the conclusions of this article will be made available by the authors, without undue reservation.

## References

[B1] Abubakar U. Awaisu A. Khan A. H. Alam K. (2023). Impact of COVID-19 pandemic on healthcare-associated infections: a systematic review and meta-analysis. Antibiotics 12, 1600. doi: 10.3390/antibiotics12111600 37998802 PMC10668951

[B2] Amarsy R. Jacquier H. Munier A. L. Merimèche M. Berçot B. Mégarbane B. (2021). Outbreak of NDM-1-producing Klebsiella pneumoniae in the intensive care unit during the COVID-19 pandemic: Another nightmare. Am. J. Infect. Control 49, 1324–1326. doi: 10.1016/j.ajic.2021.07.004 34273465 PMC8279923

[B3] Ayoub Moubareck C. Hammoudi Halat D. (2022). The collateral effects of COVID-19 pandemic on the status of carbapenemase-producing pathogens. Front. Cell. Infect. Microbiol. 12, 823626. doi: 10.3389/fcimb.2022.823626 35372126 PMC8968076

[B4] Bedford J. Farrar J. Ihekweazu C. Kang G. Koopmans M. Nkengasong J. (2019). A new twenty-first century science for effective epidemic response. Nature 575, 130–136. doi: 10.1038/s41586-019-1717-y 31695207 PMC7095334

[B5] Biedrzycka M. Urbanowicz P. Izdebski R. Literacka E. Hryniewicz W. Gniadkowski M. . (2021). Dissemination of klebsiella pneumoniae ST147 NDM-1 in Poland, 2015–19. J. Antimicrob. Chemother. 76, 2538–2545. doi: 10.1093/jac/dkab207 34164678

[B6] Caliskan-Aydogan O. Alocilja E. C. (2023). A review of carbapenem resistance in Enterobacterales and its detection techniques. Microorganisms 11, 1491. doi: 10.3390/microorganisms11061491 37374993 PMC10305383

[B7] Centers for Disease Control and Prevention (2026). NHSN’s Guide to the 2022 Baseline Standardized Infection Ratios (Atlanta: CDC).

[B8] Choiński M. Wasiewicz-Ciach P. Marzec M. T. Kuczyński P. Marszałek A. Wydra-Rojek A. . (2024). The issue of antibiotic-resistant bacterial infections in intensive care units (ICUs) – epidemiology, risk factors and prevention. Literature review. Qual. Sport 20, 53946. doi: 10.12775/QS.2024.20.53946

[B9] European Centre for Disease Prevention and Control (2025). Protocol for the Surveillance of Healthcareassociated Infections and Prevention Indicators in European Intensive Care Units (Stockholm: ECDC).

[B10] Fauci A. S. Lane H. C. Redfield R. R. (2020). Covid-19 - navigating the uncharted. N. Engl. J. Med. 382, 1268–1269. doi: 10.1056/nejme2002387 32109011 PMC7121221

[B11] Filipkowska M. Orzechowska M. Zarychta M. Cybulski M. (2025). Epidemiological situation of antibiotic-resistant microorganisms identified in patients hospitalised at the University Teaching Hospital in Bialystok, Poland, in the 2020-2023 period. Antibiotics 14, 1128. doi: 10.3390/antibiotics14111128 41301623 PMC12649106

[B12] Frérot M. Lefebvre A. Aho S. Callier P. Astruc K. Aho Glélé L. S. (2018). What is epidemiology? Changing definitions of epidemiology 1978-2017. PloS One 13, e0208442. doi: 10.1371/journal.pone.0208442 30532230 PMC6287859

[B13] Golli A. L. Popa S. G. Ghenea A. E. Turcu F. L. (2025). The impact of the COVID-19 pandemic on the antibiotic resistance of Gram-negative pathogens causing bloodstream infections in an intensive care unit. Biomedicines 13, 379. doi: 10.3390/biomedicines13020379 40002795 PMC11852776

[B14] Lee Y. M. Kim D. Y. Kim E. J. Park K. H. Lee M. S. (2023). Impact of COVID-19 pandemic on healthcare-associated infections at intensive care units in South Korea: data from the Korean National Healthcare-Associated Infections Surveillance System (KONIS). J. Hosp. Infect. 138, 52–59. doi: 10.1016/j.jhin.2023.05.010 37277015 PMC10239201

[B15] Lin X. C. Li C. L. Zhang S. Y. Yang X. F. Jiang M. (2023). The global and regional prevalence of hospital-acquired carbapenem-resistant Klebsiella pneumoniae infection: a systematic review and meta-analysis. Open Forum Infect. Dis. 11, ofad649. doi: 10.1093/ofid/ofad649 38312215 PMC10836986

[B16] Ma Z. Mo L. Li C. Hu J. Zheng W. Zeng S. . (2025). Epidemiology and genomic characteristics of carbapenem-resistant Klebsiella pneumoniae in intensive care unit from 2021 to 2024. BMC Microbiol. 25, 774. doi: 10.1186/s12866-025-04497-0 41287044 PMC12642057

[B17] Martin R. M. Bachman M. A. (2018). Colonization, infection, and the accessory genome of Klebsiella pneumoniae. Front. Cell. Infect. Microbiol. 8, 4. doi: 10.3389/fcimb.2018.00004 29404282 PMC5786545

[B18] McArthur D. B. (2019). Emerging infectious diseases. Nurs. Clin. North. Am. 54, 297–311. doi: 10.1016/j.cnur.2019.02.006 31027668 PMC7096727

[B19] Mercer A. J. (2018). Updating the epidemiological transition model. Epidemiol. Infect. 146, 680–687. doi: 10.1017/s0950268818000572 29557320 PMC9134371

[B20] Montrucchio G. Corcione S. Rodigari L. Barganou D. Risso C. Traversi R. . (2025). Clinical impact of New Delhi metallo-beta-lactamase-producing Enterobacterales in critically ill patients: Are we ready to face the challenge? J. Clin. Med. 14, 5688. doi: 10.3390/jcm14165688 40869516 PMC12386272

[B21] Morens D. M. Folkers G. K. Fauci A. S. (2004). The challenge of emerging and re-emerging infectious diseases. Nature 430, 242–249. doi: 10.1038/nature02759 15241422 PMC7094993

[B22] Olczak-Pienkowska A. Hryniewicz W. (2021). Impact of social, economic, and healthcare factors on the regional structure of antibiotic consumption in primary care in Poland (2013–2017). Front. Public Health 9, 680975. doi: 10.3389/fpubh.2021.680975 34395362 PMC8358207

[B23] Omran A. R. (1971). The epidemiologic transition. A theory of the epidemiology of population change. Bull. World Health Organ. 79, 161–170. doi: 10.2307/3349375 11246833 PMC2566347

[B24] Peconi C. Martini E. Sarti D. Prospero E. (2025). Impact of the COVID-19 pandemic on healthcare-associated infections and multidrug-resistant microorganisms in Italy: a systematic review. J. Infect. Public Health 18, 102729. doi: 10.1016/j.jiph.2025.102729 40056892

[B25] Sakagianni A. Koufopoulou C. Koufopoulos P. Feretzakis G. Koumaki V. (2025). The impact of COVID-19 on the epidemiology of carbapenem resistance. Antibiotics 14, 916. doi: 10.3390/antibiotics14090916 41009895 PMC12466860

[B26] Santosa A. Wall S. Fottrell E. Högberg U. Byass P. (2014). The development and experience of epidemiological transition theory over four decades: a systematic review. Glob. Health Action 7, 23574. doi: 10.3402/gha.v7.23574 24848657 PMC4038769

[B27] Shih-Hao L. Chun-Yu L. Ching-Tzu H. Jyun-Ji H. Po-Liang L. (2021). The impact of universal face masking and enhanced hand hygiene for COVID-19 disease prevention on the incidence of hospital-acquired infections in a Taiwanese hospital. Int. J. Infect. Dis. 104, 15–18. doi: 10.1016/j.ijid.2020.12.072 33383221 PMC7832929

[B28] Tacconelli E. Carrara E. Savoldi A. Harbarth S. Mendelson M. Monnet D. L. . (2018). Discovery, research, and development of new antibiotics: the WHO priority list of antibiotic-resistant bacteria and tuberculosis. Lancet Infect. Dis. 18, 318–327. doi: 10.1016/s1473-3099(17)30753-3 29276051

[B29] Tenderenda A. Łysakowska M. E. Gawron-Skarbek A. (2023). The prevalence of alert pathogens and microbial resistance mechanisms: a three-year retrospective study in a general hospital in Poland. Pathogens 12, 1401. doi: 10.3390/pathogens12121401 38133286 PMC10746124

[B30] Wee L. E. I. Conceicao E. P. Tan J. Y. Magesparan K. D. Amin I. B. M. Ismail B. B. S. . (2021). Unintended consequences of infection prevention and control measures during COVID-19 pandemic. Am. J. Infect. Control 49, 469–477. doi: 10.1016/j.ajic.2020.10.019 33157180 PMC7610096

[B31] Weiner-Lastinger L. M. Pattabiraman V. Konnor R. Y. Patel P. R. Wong E. Xu S. Y. . (2022). The impact of coronavirus disease 2019 (COVID-19) on healthcare-associated infections in 2020: A summary of data reported to the National Healthcare Safety Network. Infect. Control Hosp. Epidemiol. 43, 12–25. doi: 10.1017/ice.2021.362 34473013

[B32] World Health Organization (2014). Antimicrobial Resistance: Global Report on Surveillance (Geneva: World Health Organization), 35–40.

[B33] World Health Organization (2017). Prioritization of Pathogens to Guide Discovery, Research and Development of New Antibiotics for Drug-Resistant Bacterial Infections, Including Tuberculosis (Geneva: World Health Organization).

[B34] Yao Y. Zha Z. Li L. Tan H. Pi J. You C. . (2024). Healthcare-associated carbapenem-resistant Klebsiella pneumoniae infections are associated with higher mortality compared to carbapenem-susceptible K. pneumoniae infections in the intensive care unit: a retrospective cohort study. J. Hosp. Infect. 148, 30–38. doi: 10.1016/j.jhin.2024.03.003 38513959

